# Non-invasive predictors for infranodal conduction delay in patients with left bundle branch block after TAVR

**DOI:** 10.1007/s00392-021-01924-w

**Published:** 2021-08-26

**Authors:** Chloé Auberson, Patrick Badertscher, Antonio Madaffari, Meriton Malushi, Luc Bourquin, Florian Spies, Stefanie Aeschbacher, Gregor Fahrni, Christoph Kaiser, Raban Jeger, Stefan Osswald, Christian Sticherling, Sven Knecht, Michael Kühne

**Affiliations:** Division of Cardiology, University of Basel Hospital, Petersgraben 4, 4031 Basel, Switzerland

**Keywords:** LBBB, TAVI, TAVR, Conduction delay, Electrocardiogram, ECG

## Abstract

**Aims:**

Left bundle branch block (LBBB) is the most common conduction disorder after transcatheter aortic valve replacement (TAVR) with an increased risk of atrioventricular (AV) block. The aim of the current study was to identify non-invasive predictors for infranodal conduction delay in patients with LBBB.

**Methods:**

We analyzed consecutive patients undergoing TAVR with pre-existing or new-onset LBBB between August 2014 and August 2020. His ventricular (HV) interval measurement was performed on day 1 after TAVR. Baseline, procedural, as well as surface and intracardiac electrocardiographic parameters were included. Infranodal conduction delay was defined as HV interval > 55 ms.

**Results:**

Of 825 patients screened after TAVR, 151 patients (82 ± 6 years, 39% male) with LBBB were included. Among these, infranodal conduction delay was observed in 25%. ΔPR (difference in PR interval after and before TAVR), PR and QRS duration after TAVR were significantly longer in the group with HV prolongation. In a multivariate analysis in patients with sinus rhythm (*n* = 123), ΔPR (OR per 10 ms increase: 1.52; 95%CI: 1.19–2.01; *p* = 0.002) was the only independent factor associated with infranodal conduction delay. A change in PR interval by 20 ms yielded a specificity of 83% and a sensitivity of 46%, with a negative predictive value of 84% and a positive predictive value of 45% to predict HV prolongation.

**Conclusions:**

Simple analysis of surface ECG and a calculated ΔPR < 20 ms can be used as predictor for the absence of infranodal conduction delay in post-TAVR patients with LBBB.

**Graphical abstract:**

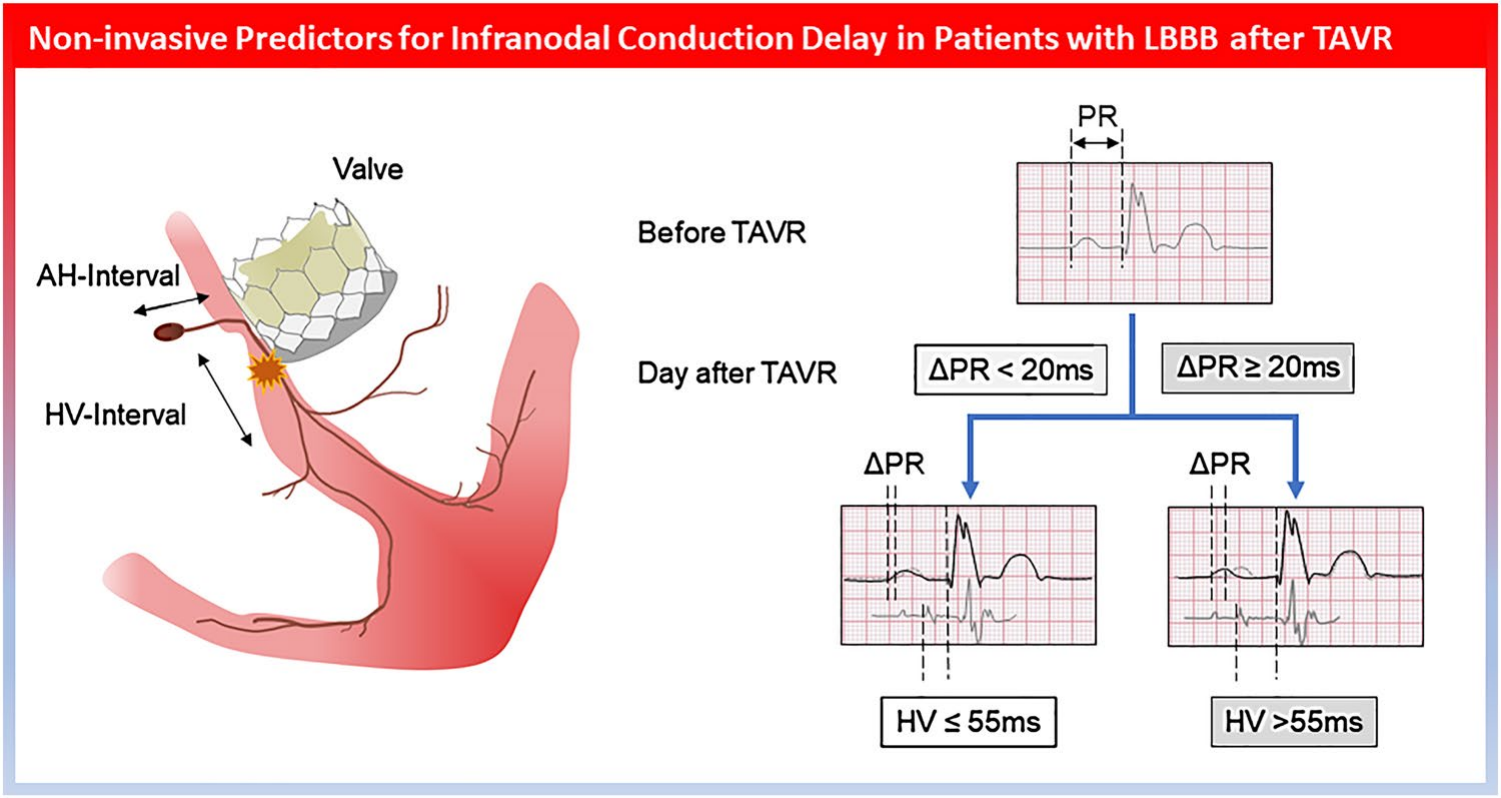

**Supplementary Information:**

The online version contains supplementary material available at 10.1007/s00392-021-01924-w.

## Introduction

Transcatheter aortic valve replacement (TAVR) has become a well-established treatment option for patients with symptomatic aortic stenosis with an intermediate-to-high surgical risk [1–4]. Periprocedural complications of TAVR have decreased with improved operator experience as well as newer transcatheter heart valve technologies [5, 6]. However, conduction disturbances, in general, and left bundle branch block (LBBB), in particular, remain a common problem after TAVR, due to the anatomical proximity of the conduction system to the aortic valve [7]. Despite the relatively high incidence of LBBB ranging between 4 and 65% [6], its management is still ambiguous. It is known that patients with new-onset LBBB after TAVR have an increased risk of all-cause mortality, cardiovascular mortality, rehospitalization and progression to complete atrioventricular block [5, 8, 9]. Simplified algorithms for the treatment of conduction disturbance after TAVR were proposed based on non-invasive electrocardiographic (ECG) measurements in a recent expert panel document [1, 10]. For selective patients with pre-existing conduction disturbance as well as new-onset LBBB, an invasive electrophysiological study (EPS) may be conducted to assess the risk of high-degree atrioventricular conduction block (HAVB) based on infranodal conduction and consequently guide the decision for permanent pacemaker implantation (PPI) [10–12]. Recently, an EPS-tailored strategy was proposed to stratify patients with LBBB after TAVR regarding the development of HAVB based on a His ventricular (HV) interval cutoff of > 55 ms [13].

The objective of this study was to identify clinical and ECG predictors of infranodal conduction disturbance (defined as HV interval > 55 ms) in patients with LBBB after TAVR. This may aid clinical decision making in patients with LBBB after TAVR.

## Methods

### Study design and patient population

We analyzed the data from patients collected in the prospective Swiss TAVR registry (NCT01368250) for the period from August 2014 to August 2020, treated at our institution. Written informed consent was obtained from all patients and the study was approved by the local ethics committee.

Patients included in the present analysis were those with pre-existing or new-onset LBBB after TAVR. The clinical and procedural characteristics were obtained from the electronic patient medical records. Exclusion criteria were TAVR performed through other than transfemoral implantation route, valve-in-valve implantation, previous PPI, and HAVB after TAVR requiring PPI, and missing electrophysiological (EP) measurement of the HV interval. Valve types included in our study were self‐expandable Evolut R and Evolut R Pro (Medtronic, Minneapolis, MN), Portico (St. Jude Medical, St Paul, MN), Acurate NEO (Boston Scientific, Natick, MA), balloon‐expandable Sapien 3 (Edwards Life Science, Irvine, CA), or mechanically expandable Lotus and Lotus Edge (Boston Scientific Inc., Marlborough, MA).

#### Transcatheter aortic valve replacement

TAVR procedures were performed as previously described [13]. Briefly, transthoracic echocardiography, coronary angiography, and ECG-triggered multislice computed tomography scan of the aorta were performed for procedural planning. The implantation of the valve was performed according to the recommendations of the manufacturer. During the procedure, a temporary pacemaker using a quadripolar catheter (5Fr, CRD, St Jude Medical, USA) was positioned in the right ventricular apex. After implantation, patients were transferred to the intensive care unit overnight with the temporary pacemaker left in place and programmed to VVI 30 bpm in case of HAVB. Continuous rhythm monitoring by telemetry was performed for 72 h.

### Electrocardiographic assessment

The 12-lead electrocardiograms obtained with a standard ECG recorder (Schiller, Baar, Switzerland) prior to and during hospital stay were analyzed. Each ECG recording was assessed for rhythm and conduction disturbance with a sweep speed of 25 mm/s and standard augmentation of 1 mV/10 mm. First-degree atrioventricular block was defined as a PR interval ≥ 200 ms. LBBB was defined using conventional criteria with a QRS duration ≥ 120 ms, an R wave peak delay in lead V5/V6 of > 60 ms, and an rS or QS in lead V1 and V2 [14]. The automatically calculated PR interval and QRS duration by the ECG recorder were included in the analysis. To improve the accuracy of the measurements, each ECG was manually reviewed, and if necessary, corrected.

We defined the difference of the PR interval and the QRS duration before and after the procedure as ΔPR and ΔQRS, respectively. To be able to use PR interval for prediction models, only patients with sinus rhythm were included. Patients with atrial fibrillation were included in a second analysis.

### Electrophysiology study after TAVR

In all patients showing LBBB on the ECG the day after the procedure, we performed a limited EPS [13]. In brief, intracardiac measurements were obtained using the quadripolar diagnostic catheter (5F, CRD, St. Jude Medical) used as a temporary pacemaker wire during TAVR. After withdrawal of the catheter from the ventricle to the His position, HV interval was measured over three consecutive beats using the electronic calipers with a sweep speed of 100 mm/s on the EP system (Sensis, Siemens, Germany). Simultaneously, PR and QRS durations were obtained. Based on these measurements, patients were stratified into a group with normal HV interval (HV ≤ 55 ms) and with prolonged HV interval (HV > 55 ms).

### Follow-up

Follow-up was performed 3 months after TAVI, including physical examination, 12-lead ECG and transthoracic echocardiography. In patients with pacemaker implantation due to prolonged HV interval, pacemaker interrogation was performed. No need for pacing was defined as < 1% ventricular pacing and intrinsic 1:1 AV conduction with the device programmed to VVI 30 bpm [13].

### Statistical analysis

Continuous variables are presented as mean and standard deviation or median (interquartile range) and categorical variables as numbers and percentages. *T* test was used for continuous, normally distributed and the Wilcoxon test for skewed variables. Categorical variables were compared using Chi-square or the Fisher’s exact test as appropriate.

Using logistic regression models, we first performed a univariate analysis to identify unadjusted associations between baseline data, procedural characteristics, ECG parameters and the HV prolongation > 55 ms. Potential predictors with a *p* value ≤ 0.05 were selected for multivariable analysis and subsequently corrected for patient age, sex and body surface area.

Receiver-operating characteristic (ROC) curves were generated and the area under the curve (AUC) was calculated for uni- and multivariate analyses. The optimal thresholds (cutoffs) to predict a prolonged HV interval (HV > 55 ms) were determined based on the Youden’s index [15] as well as for an easy and clinically applicable cutoff of 10 ms and 20 ms. All statistical analyses were performed using R version 4.0.2 (R Foundation for statistical computing, Vienna, Austria).

## Results

### Baseline data

**Fig. 1:**
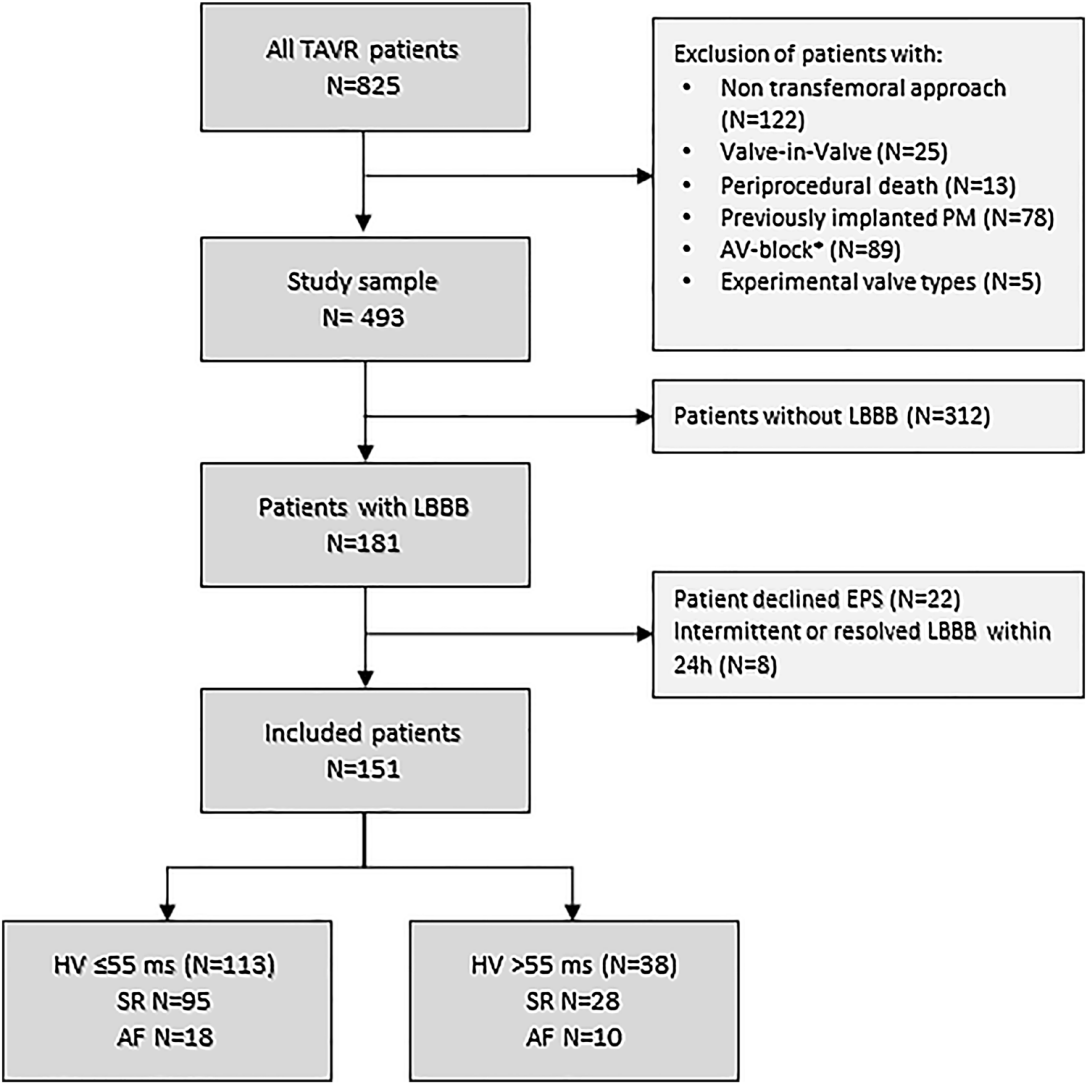
Flowchart of the total cohort. *AF *atrial fibrillation, *EPS *electrophysiological study, *HV* His ventricular, *LBBB *left bundle branch block, *PM* pacemaker, *SR* sinus rhythm, *TAVR* transfemoral aortic valve replacement. *Patient with higher-grade AVB underwent pacemaker implant directly after TAVR

After exclusion of 332 of the 825 patients based on the described criteria, 181 of the 493 (37%) patients remaining showed LBBB the day after the procedure (Fig. 1). After exclusion of patients declining EPS (*n* = 22) and patients with intermittent LBBB (*n* = 8), the final cohort available for analysis consisted of 151 patients. The mean age was 82 ± 6 years, 39% of patients were male (Table 1). The median gradient across the aortic valve was 48 (IQR 39; 59) mmHg, the mean aortic valve area was 0.7 ± 0.2 cm^2^. On EPS 1 day after TAVR, a prolonged HV interval was detected in 25% of patients and a normal HV interval was detected in 75% of patients.

**Table 1:** Clinical and procedural characteristics

Parameter	Overall (*n* = 151)	HV ≤ 55 ms (*n* = 113)	HV > 55 ms (*n* = 38)	*p*-value
Age, years	82 ± 6	82 ± 6	84 ± 6	0.067
Male sex	59 (39%)	39 (34%)	20 (53%)	0.074
Height, cm	166 ± 8	165 ± 8	168 ± 9	0.123
Weight, kg	71 (63; 86)	70 (64; 83)	71 (62; 90)	0.363
Body surface, m^2^	1.85 ± 0.25	1.82 ± 0.22	1.92 ± 0.31	0.041
Hypertension	114 (76%)	90 (80%)	24 (63%)	0.068
CAD	73 (48%)	52 (46%)	21 (55%)	0.424
Dyslipidemia	81 (54%)	63 (56%)	18 (47%)	0.479
Diabetes	41 (27%)	33 (29%)	8 (21%)	0.443
Prior myocardial infarction	26 (17%)	22 (20%)	4 (11%)	0.310
Prior stroke	21 (14%)	15 (13%)	6 (16%)	0.907
Angina pectoris	52 (35%)	40 (36%)	12 (32%)	0.764
Atrial fibrillation	59 (39%)	38 (34%)	21 (55%)	0.030
NYHA				
I	15 (10%)	10 (9%)	5 (14%)	0.613
II	57 (38%)	45 (40%)	12 (32%)	0.543
III	69 (46%)	51 (45%)	18 (49%)	0.855
IV	9 (6%)	7 (6%)	2 (5%)	1.000
Previous cardiac surgery	12 (8%)	8 (7%)	4 (11%)	0.739
Medication				
Beta-blockers	77 (51%)	57 (50%)	20 (53%)	0.963
Class Ic	1 (1%)	1 (1%)	0	1.000
Class III	12 (8%)	2 (2%)	10 (26%)	< 0.001
Pre-procedural echocardiography				
DPmean, mmHg	48 (39; 59)	48 (39; 60)	48 (39; 55)	0.785
Aortic valve area, cm^2^	0.7 ± 0.2	0.7 ± 0.2	0.8 ± 0.2	0.217
LVEF, %	59 (45; 60)	55 (45; 60)	60 (45; 61)	0.488
Valve type				
Balloon-expandable				
Sapien 3	20 (13%)	14 (13%)	6 (16%)	0.796
Self-expandable				
CoreValve	3 (2%)	2 (2%)	1 (3%)	1.000
Evolut R	5 (3%)	4 (4%)	1 (3%)	1.000
Evolut Pro	21 (14%)	13 (12%)	8 (21%)	0.230
Portico	50 (33%)	42 (37%)	8 (21%)	0.104
Symetis Acurate Neo	18 (12%)	13 (12%)	5 (13%)	1.000
Mechanical-expandable				
Lotus	30 (20%)	21 (19%)	9 (24%)	0.655
Lotus Edge	4 (3%)	4 (4%)	0 (0%)	0.554

Data are presented as mean ± SD or median (interquartile range) for continuous variables and as *n* (%) for categorical variables

*BMI *body mass index, *CAD *coronary artery disease, *DPmean *mean transvalvular pressure gradient, *LBBB* left bundle branch block, *LVEF *left ventricular ejection fraction, *NYHA *New York Heart Association

The demographic and pre-procedural echocardiographic parameters were comparable between patients with normal and prolonged HV interval except an increased body surface area (1.92 ± 0.31 versus 1.82 ± 0.22; *p* = 0.041) and a higher prevalence of diagnosis of atrial fibrillation (55 versus 34%; *p* = 0.030) in the group with a prolonged HV interval. No differences between the two groups were observed for the valve types implanted. Exclusion of patients with pre-existing LBBB (*n* = 22) showed comparable results (Supplement 1). The median PR interval and QRS duration before TAVR were normal in both groups. ECG parameters before and after TAVR are listed in Table 2.

**Table 2:** ECG findings before and after TAVR

	Overall (*n* = 151)	≤ 55 ms (*n* = 113)	> 55 ms (*n* = 38)	
Baseline ECG				
LBBB	22 (15)	16 (15)	6 (16)	0.794
LAFB	11 ( 7)	4 ( 4)	7 (18)	0.006
AVB I	34 (23)	26 (24)	8 (21)	0.825
PR interval, ms	178 (157; 203)	175 (156; 204)	184 (174; 200)	0.203
QRS duration, ms	97 (88; 110)	96 (87; 112)	99 (91; 108)	0.262
Post-TAVR ECG				
PR interval, ms	197 (170; 218)	185 (165; 211)	216 (197; 236)	< 0.001
QRS duration, ms	146 (138, 156)	145 (136; 152)	153 (144; 161)	0.004
ΔPR, ms	9 (2; 18)	7 (0; 14)	16 (11; 33)	< 0.001
ΔQRS, ms	48 (32; 60)	46 (32; 59)	52 (31; 67)	0.231

Data are presented as mean ± SD or median (interquartile range) for continuous variables and as *n* (%) for categorical variables

*AVB I* AV block I; LAFB—left anterior fascicular block, *LBBB *left bundle branch block, *TAVR* transfemoral aortic valve replacement. ΔPR could not be measured in *n* = 20 patients because of AF in either ECG

### Follow-up data

After exclusion of 7 of the 38 patients with prolonged HV interval (3 patients rejected PPI, 1 death before follow-up due to non-cardiac causes, and 3 patients without device interrogation showing pacing percentages), 19 of the 31 patients (61%) showed a need for pacing ≥ 1%. Of the 113 patients without HV prolongation and LBBB after TAVR, four (4%) showed an indication for PPI based on a documented episode of HAVB (three of four from implantable loop recorders (ILR)). During follow-up, however, none of the patients with ILR documented HAVB showed need for pacing (0%) within 3 months of follow-up.

### Predictors of prolonged HV interval

In the 123 patients with sinus rhythm during EPS, ΔPR values (odds ratio (OR) per 10 ms increase: 1.49; 95% confidence interval (CI): 1.19–1.93; *p* = 0.001), the PR interval (OR per 10 ms increase: 1.27; 95% CI: 1.11–1.48; *p* = 0.001) and QRS duration after TAVR (OR per 10 ms increase: 1.37; 95% CI: 1.03–1.84; *p* = 0.031) were significantly associated with prolonged HV interval. After correction for age, sex and body surface area in the multivariate analysis, ΔPR (OR per 10 ms increase: 1.52; 95% CI: 1.19–2.01; p = 0.002) was the only parameter associated with prolonged HV interval (Table 3). Additional correction for hypertension and a clinical diagnosis of AF (but with sinus rhythm before and after TAVR) did not result in relevant changes of our model (OR per 10 ms increase of ΔPR 1.41; 95% CI: 1.09–1.88; *p* = 0.013) (Supplement 2).

**Table 3:** Univariate and multivariate predictors for prolonged hv interval of patients in sinus rhythm

	Univariate OR (95%CI)	*p* Value	Multivariate OR (95%CI)	*p* Value
Age, years	1.04 (0.96–1.13)	0.328	1.06 (0.97–1.18)	0.224
Male sex	2.89 (1.23–6.99)	0.016	2.26 (0.79–6.68)	0.131
Height, cm	1.04 (0.99–1.10)	0.128		
Weight, kg	1.03 (1.00–1.05)	0.027		
Body surface area, per 0.1m^2^	1.25 (1.05–1.51)	0.016	1.26 (1.01–1.60)	0.046
Hypertension	0.54 (0.22–1.38)	0.187		
CAD	1.72 (0.73–4.15)	0.217		
Dyslipidemia	1.01 (0.43–2.41)	0.977		
Diabetes mellitus	0.64 (0.20–1.77)	0.420		
Prior myocardial infarction	0.62 (0.17–1.85)	0.430		
Prior stroke	1.50 (0.44–4.52)	0.484		
Stable angina pectoris	0.92 (0.37–2.19)	0.854		
AF	2.43 (0.97–5.99)	0.055		
NYHA				
I				
II	0.56 (0.15–2.41)	0.409		
III	0.59 (0.16–2.50)	0.443		
IV	1.12 (0.12–8.78)	0.911		
Previous cardiac surgery	2.16 (0.42–9.43)	0.314		
Preinterventional echocardiography				
DPmean, mmHg	0.99 (0.97–1.02)	0.681		
Aortic valve area, per 0.1 mm^2^	1.16 (0.92–1.47)	0.208		
LVEF, %	1.01 (0.97–1.04)	0.726		
Baseline ECG				
PR, per 10 ms	1.09 (0.96–1.25)	0.174		
QRS, per 10 ms	1.03 (0.87–1.21)	0.706		
ECG after TAVR				
PR, per 10 ms	1.27 (1.11–1.48)	0.001		
QRS, per 10 ms	1.37 (1.03–1.84)	0.031		
Comparison of ECG before and After TAVR				
ΔPR, per 10 ms	1.49 (1.19–1.93)	0.001	1.52 (1.19–2.01)	0.002
ΔQRS, per 10 ms	1.09 (0.91–1.31)	0.345		

*BMI *body mass index, *CAD *coronary artery disease, *DPmean *mean transvalvular pressure gradient, *CI* confidence interval, *LBBB* left bundle branch block, *LVEF *left ventricular ejection fraction, *NYHA *New York Heart Association, *OR* odds ratio

When including patients with atrial fibrillation on the ECG before and after TAVR in the analysis (*n* = 151), the QRS width post-TAVR (OR per 10 ms increase: 1.46; 95% CI: 1.13–1.93; *p* = 0.006), but not the ΔQRS (OR per 10 ms increase: 0.95–1.32; *p* = 0.196) was identified as a predictor of HV prolongation. After correction for sex, age, body surface area and class III antiarrhythmic therapy, QRS post-TAVR (OR: 1.34; 95% CI: 1.02–1.79; p = 0.041) remained a significant predictor in the multivariate analysis (Supplement 3).

**Fig. 2:**
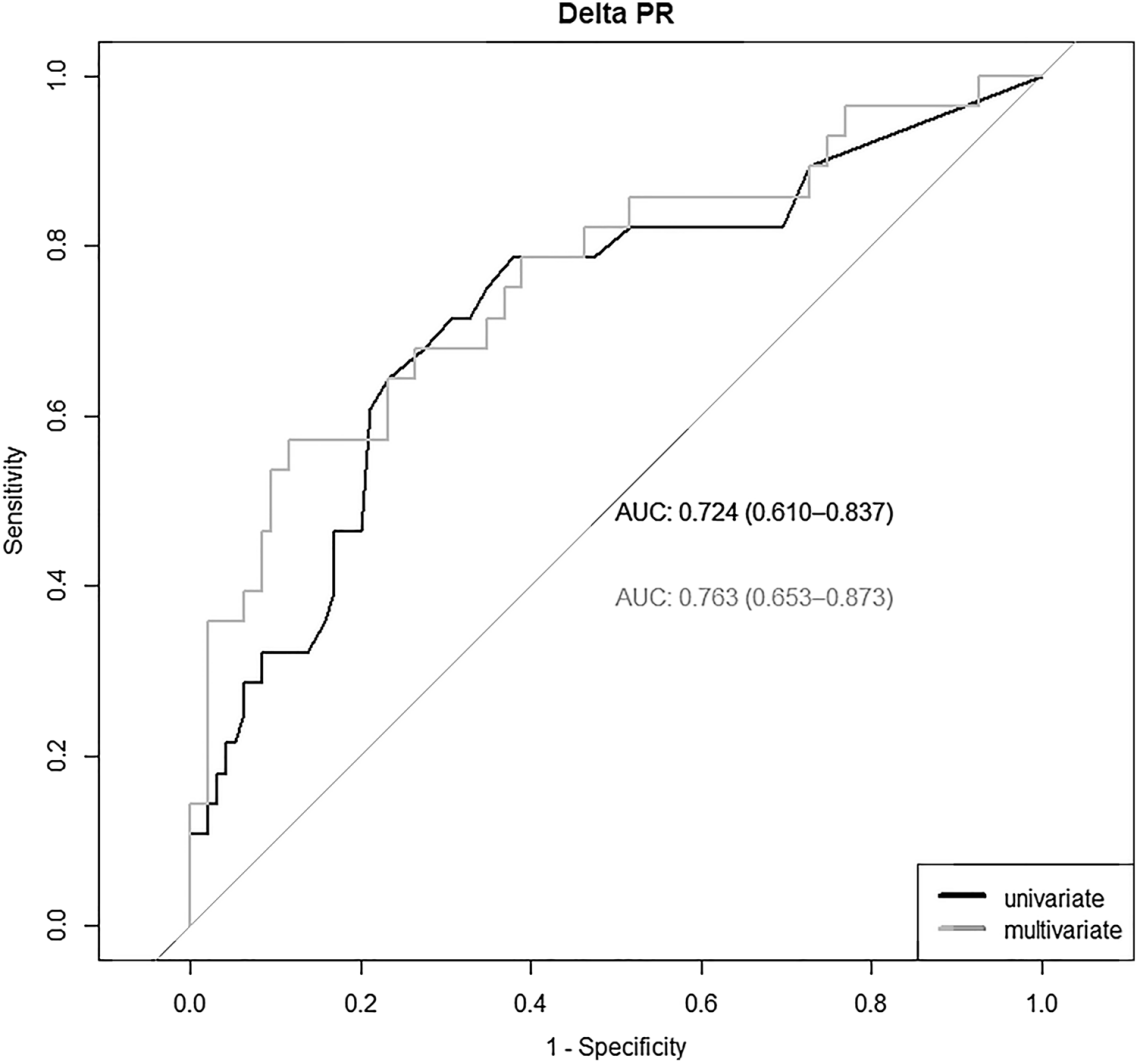
Receiver-operating characteristics curve demonstrating the uni- and multivariate accuracy of ΔPR for predicting prolonged His ventricular measurement. AUC indicates area under the curve

The AUC for the ROC curve for ΔPR to predict a prolonged HV was 0.724 (95% CI: 0.610–0.837) in the univariate and 0.763 (95% CI: 0.653–0.873) in the multivariate analysis corrected for age, sex and body surface area, respectively (Fig. 2).

With a cutoff of ≥ 10 ms or ≥ 16 ms for ΔPR chosen based on the criteria of a clinical applicable cutoff and the Youden’s index, the sensitivity and specificity to predict HV prolongation was 79% and 62% and 61% and 79%, respectively. The positive (PPV) and negative predictive value (NPV) was 38% and 91% for a ΔPR of ≥ 10 ms or 46 and 87% for a ΔPR of ≥ 16 ms, respectively. The recently recommended clinically relevant ΔPR value of ≥ 20 ms [1] yielded a sensitivity of 46% and specificity of 83% with a PPV of 45% and NPV of 84% to predict HV prolongation.

## Discussion

The main findings of our study are: (1) prolongation of infranodal conduction delay (HV interval > 55 ms) in patients with LBBB after TAVR is frequent (25%). (2) PR interval, QRS duration and ΔPR were significantly longer after TAVR in the group with prolonged HV interval compared to the group with normal HV interval. (3) ΔPR was identified as the only independent predictor for HV prolongation with an OR 1.49 (95% CI 1.19–1.93) per increase of 10 ms in the univariate analysis. (4) The AUC of the ROC curve was 0.724 (95% CI) for ΔPR. (5) Finally, the absence of a relevant increase in PR interval (< 20 ms) rules out a critical HV interval prolongation (> 55 ms) with an NPV of 84%.

Conduction disturbances and specifically LBBB are the most common complications after TAVR [16]. LBBB has been shown to be associated with a high rate of complete AV block (20%) and syncope (16%) during the first year after TAVR compared to < 1% in patients without LBBB [8]. Due to the lack of clear guidelines, individual treatment strategies are currently implemented. A recent expert consensus decision document states that EPS (and PPI) should be considered in patients with new, progressive, or pre-existing conduction disturbance changing after the procedure [10]. In addition, in patients with persistent new-onset LBBB the day after TAVR, an invasive EPS to guide the decision for PPI was proposed in case of defined ECG changes (PR interval > 240 ms or QRS duration > 150 ms) by a scientific expert panel [1]. In pre-existing LBBB, it is recommended to consider EPS in case additional ECG changes after TAVR are observed, defined as further increase (> 20 ms) in QRS duration or PR interval, a QRS duration > 150 ms, or a PR interval > 240 ms [1]. However, evidence for these proposed cutoffs of the QRS duration and PR interval is scarce in LBBB patients after TAVR and their value to predict HAVB is unclear. The presence of an infranodal conduction delay, defined as a prolonged HV interval, can be measured in an EPS and has been shown to correlate with the development of HAVB. However, since EPS after TAVR is not readily available in all centers, the identification of non-invasive predictors for prolonged HV interval based surface ECG parameters (PR interval and QRS duration) is of high clinical relevance.

### Value of HV measurements to predict HAVB

The first description of the impact of the HV interval measurements on the prevalence of HAVB in patients with chronic LBBB was reported almost three decades ago [17]. They observed that an HV interval ≥ 70 ms was an independent predictor for progression to HAVB in patients with LBBB. More recently, several studies implemented a predefined HV cutoff after TAVR to trigger for PPI in general. The value for the HV interval range between 55 ms [12, 13], over 65 ms [18], 70 ms [19], 75 ms [11, 20], 80 ms [21], up to 100 ms [22]. Whether prophylactic PPI in the setting of prolonged HV interval improves outcomes (e.g., HAVB, syncope, hospitalizations, mortality) has not been assessed these studies and warrants further investigation in a randomized study.

Rivard et al. [18] showed a strong association of the post-procedural HV interval with HAVB. In their study, an HV interval ≥ 65 ms predicted HAVB with 83.3% sensitivity and 81.6% specificity and 82% NPV and 62% PPV. Similarly, we showed with an HV interval cutoff > 55 ms a 67% sensitivity, 84% specificity, and 90% NPV and 53% PPV [13]. In a smaller study by Mirolo et al. [19], three of five patients (60%) with PPI due to new-onset LBBB and an HV interval > 70 ms had significant ventricular pacing during follow-up of 2–4 months, reflecting episodes of HAVB. This need for pacing was similar to our observations with an HV cutoff of 55 ms.

In general, the timing of EPS may play an important factor, since the conduction behavior after TAVR might change, especially within the first 24 h [10]. Furthermore, the behavior may be different for self-expandable compared to balloon-expandable valves [23]. In this study, EPS was performed the day after TAVR and it is unclear whether EPS immediately after TAVR would yield similar results.

Since EPS is not universally available, the relationship between the intracardiac measurements (HV interval) and easily accessible clinical and surface ECG parameters is of high clinical importance. Readily available parameters that could replace the HV interval measurement may simplify clinical decision making. In our analysis, we identified the ΔPR interval as an indicator for increased risk of HV prolongation. This suggests that a simple analysis of the dynamic changes in the ECG post-TAVR could identify patients at risk for developing HAVB. This observation is in line with a previous study analyzing the occurrence of delayed AVB (> 48 h) after TAVR. [24] Significant widening of the QRS and PR interval in patients within 48 h after TAVR was observed; however, only ΔPR proved to be an independent predictor for delayed AVB in a multivariate analysis. At the other end of the spectrum, and potentially more relevant for clinical practice and streamlining workflow, in our study, the absence of a relevant increase in PR interval (< 20 ms) was able to make HV interval prolongation unlikely with a negative predictive value of 84%.

## Study limitations

This was a single-center retrospective study focusing on LBBB after TAVR. Other conduction disturbances such as RBBB and fascicular block in conjunction with RBBB were not studied. Furthermore, numerous valve types were included. However, when analyzing subgroups of self-expandable (CoreValve, Evolut R, Evolut Pro, Portico, Acurate NEO, *n* = 97), balloon-expandable (Sapien 3, *n* = 20) and mechanical-expandable valves (Lotus, Lotus Edge; *n* = 34), ΔPR was still identified as a significant predictor for HV prolongation (Supplement 4). The predictor ΔPR was only available for patients in sinus rhythm after TAVR because PR interval cannot be determined in AF. This problem could be circumvented by cardioversion, but this is not performed in clinical routine. However, when including the 20 patients in AF (13%) and consequently without PR interval as covariate, ΔQRS was the only significant predictor of a prolonged HV interval in a univariate analysis, but not after correction for other confounders in a multivariate analysis. Therefore, invasive EPS for HV interval assessment might play a more important role in patients with AF on ECG before and after TAVR. Finally, interobserver variability can be significant with ECG interpretation.

## Conclusion

Simple surface ECG criteria can be used to identify patients with LBBB at risk of an increased HV interval after TAVR. A prolongation of the PR interval of more than 20 ms was identified as independent predictor for a prolonged HV interval > 55 ms. In contrast, the absence of a significant increase in the PR interval (ΔPR < 20 ms) makes significant HV interval prolongation > 55 ms unlikely with a negative predictive value of 84%.

## Supplementary Information

Below is the link to the electronic supplementary material.

Supplementary file1 (DOCX 45 KB)

## Abbreviations

ECG: Electrocardiographic
EPS: Electrophysiological study
TAVR: Transcatheter aortic valve replacement
HAVB: High-degree atrioventricular conduction block
HV: His ventricular
LBBB: Left bundle branch block
PPI: Permanent pacemaker implantation
