# Supplementary material for: Non-invasive predictors for infranodal conduction delay in patients with left bundle branch block after TAVR
Source: Clin Res Cardiol. 2021 Aug 26;110(12):1967–76. doi: 10.1007/s00392-021-01924-w (PMC8639549; doi:10.1007/s00392-021-01924-w)
Supplement: Supplementary file 1 — Supplementary file1 (DOCX 45 KB) [file 392_2021_1924_MOESM1_ESM.docx]

# Supplement

**Supplement 1** Clinical, Procedural and ECG parameters of Patients with new-onset LBBB

| Parameter | Overall (n=129) | HV ≤55 ms (n=97) | HV >55 ms (n=32) | | p-value |
| --- | --- | --- | --- | --- | --- |
| Age, years | 82±6 | 82±6 | 84±6 | | 0.039 |
| Male sex | 50 (39%) | 34 (35%) | 16 (50%) | | 0.195 |
| Height, cm | 166±8 | 165±8 | 167±9 | | 0.247 |
| Weight, kg | 71 (64; 87) | 71 (64; 83) | 72 (62; 90) | | 0.455 |
| Body surface, m^2^ | 1.85±0.25 | 1.83±0.22 | 1.92±0.33 | | 0.079 |
| BMI, kg/m2 | 27±6 | 27±5 | 28±7 | | 0.336 |
| Hypertension | 100 (78%) | 82 (85%) | 18 (56%) | | 0.002 |
| CAD | 63 (49%) | 64 (47%) | 17 (53%) | | 0.722 |
| Dyslipidemia | 81 (54%) | 63 (56%) | 18 (47%) | | 0.283 |
| Diabetes | 37 (29%) | 30 (31%) | 7 (22%) | | 0.449 |
| Prior myocardial infarction | 21 (16%) | 18 (19%) | 3 (9%) | | 0.345 |
| Prior stroke | 18 (14%) | 13 (13%) | 5 (16%) | | 0.984 |
| Angina pectoris | 41 (32%) | 31 (33%) | 10 (31%) | | 1.000 |
| AF | 51 (40%) | 34 (35%) | 17 (53%) | | 0.109 |
| NYHA |  |  |  | |  |
| I | 15 (12%) | 10 (10%) | 5 (16%) | | 0.578 |
| II | 47 (37%) | 38 (40%) | 9 (29%) | | 0.420 |
| III | 58 (45%) | 43 (44%) | 15 (48%) | | 0.851 |
| IV | 8 (6%) | 6 (6%) | 2 (7%) | | 1.000 |
| Previous cardiac surgery | 8 (6%) | 5 (5%) | 3 (9%) | | 0.663 |
| **Pre-procedural echocardiography** | | | | | |
| DPmean, mmHg | 48 (39; 59) | 48 (39; 60) | 48 (39; 55) | | 0.547 |
| Aortic valve area, cm^2^ | 0.7±0.2 | 0.7±0.2 | 0.8±0.2 | | 0.263 |
| LVEF, % | 59 (45; 60) | 55 (45; 60) | 60 (45; 61) | | 0.641 |
| **Valve type** | | | | | |
| *Balloon-expandable* | | | | | |
| Sapien 3 | 18 (14%) | 13 (13%) | 5 (16%) | | 0.984 |
| *Self-expandable* | | | | | |
| CoreValve | 3 (2%) | 2 (2%) | 1 (3%) | | 1.000 |
| Evolut R | 4 (3%) | 4 (4%) | 0 (0%) | | 0.563 |
| Evolut Pro | 18 (14%) | 11 (11%) | 7 (22%) | | 0.231 |
| Portico | 42 (33%) | 36 (37%) | 6 (19%) | | 0.088 |
| Symetis Acurate Neo | 13 (10%) | 9 (9%) | 4 (13%) | | 0.852 |
| *mechanical-expandable* | | | | | |
| Lotus | 30 (20%) | 21 (19%) | 9 (24%) | | 0.366 |
| Lotus Edge | 4 (3%) | 4 (4%) | 0 (0%) | | 0.563 |
| **Baseline ECG** | | | | | |
| AVB I | 26 (21) | 22 (24) | 4 (13) | 0.213 | |
| LAHB | 10 (8) | 4 ( 4) | 6 (19) | 0.015 | |
| PR interval, ms | 178 (156; 200) | 178 (156; 204) | 182 (167; 193) | 0.829 | |
| QRS duration, ms | 94 (87 ; 102) | 92 (85; 102) | 96 (90; 102) | 0.185 | |
| **Post-TAVR ECG** | | | | | |
| PR interval, ms | 195 (171; 219) | 187 (166; 212) | 214 (192; 232) | 0.012 | |
| QRS duration, ms | 145 (136, 153) | 143 (136; 149) | 152 (143; 160) | 0.007 | |
| ΔPR, ms | 9 (3; 19) | 8 (2; 14) | 16 (13; 36) | 0.001 | |
| ΔQRS, ms | 51 (40; 62) | 51 (39; 60) | 56 (41; 70) | 0.142 | |

AF - atrial fibrillation; BMI - body mass index; CAD - coronary artery disease; DPmean - mean transvalvular pressure gradient; LBBB - left bundle branch block; LVEF - left ventricular ejection fraction; NYHA - New York Heart Association.

**Supplement 2** Multivariate logistic regression corrected for age, sex, body surface area, AF diagnosis and Hypertension in patients with sinus rhythm

|  | Multivariate OR (95%CI) | P Value |
| --- | --- | --- |
| Age, y | 1.07 (0.98-1.20) | 0.161 |
| Male sex | 2.18 (0.74-6.66) | 0.162 |
| Body surface area, per 0.1m^2^ | 1.33 (1.05-1.74) | 0.024 |
| Hypertension | 0.52 (0.17-1.62) | 0.246 |
| AF | 2.89 (0.95-8.98) | 0.061 |
| ΔPR, per 10 ms | 1.41 (1.09-1.88) | 0.013 |

N=123, AF – atrial fibrillation

**Supplement 3** Univariate and Multivariate Predictors for prolonged HV Interval of all patients (Sinus rhythm and AF and consequently without PR measurements)

|  | Univariate OR (95%CI) | P Value | Multivariate OR (95%CI) | P Value |
| --- | --- | --- | --- | --- |
| Age, years | 1.06 (1.00-1.14) | 0.069 | 1.11 (1.03-1.22) | 0.011 |
| Male sex | 2.11 (1.00-4.48) | 0.050 | 1.80 (0.72-4.54) | 0.209 |
| Height, cm | 1.04 (0.99-1.09) | 0.125 |  |  |
| Weight, kg | 1.02 (1.00-1.04) | 0.068 |  |  |
| Body surface area, per 0.1m^2^ | 1.16 (1.00-1.35) | 0.045 | 1.20 (0.99-1.49) | 0.070 |
| Hypertension | 0.44 (0.20-0.99) | 0.044 |  |  |
| CAD | 1.45 (0.69-3.06) | 0.325 |  |  |
| Dyslipidemia | 0.71 (0.34-1.49) | 0.371 |  |  |
| Diabetes mellitus | 0.65 (0.25-1.50) | 0.331 |  |  |
| Prior myocardial infarction | 0.49 (0.14-1.39) | 0.214 |  |  |
| Prior stroke | 1.22 (0.41-3.30) | 0.699 |  |  |
| Stable angina pectoris | 0.82 (0.36-1.77) | 0.619 |  |  |
| AF | 2.44 (1.16-5.21) | 0.020 |  |  |
| NYHA |  |  |  |  |
| I |  |  |  |  |
| II | 0.53 (0.16-1.98) | 0.324 |  |  |
| III | 0.71 (0.22-2.52) | 0.570 |  |  |
| IV | 0.57 (0.07-3.56) | 0.564 |  |  |
| Previous cardiac surgery | 1.54 (0.39-5.23) | 0.500 |  |  |
| Preinterventional echocardiography | | | | |
| DPmean, mmHg | 1.00 (0.98-1.02) | 0.933 |  |  |
| Aortic valve area, per 0.1mm^2^ | 2.92 (0.53-16.63) | 0.218 |  |  |
| LVEF, % | 1.01 (0.98-1.04) | 0.623 |  |  |
| Baseline ECG | | | | |
| PR, per 10 ms | n.A. | n.A. |  |  |
| QRS, per 10 ms | 1.03 (0.88-1.20) | 0.678 |  |  |
| Post-TAVR ECG | | | | |
| PR, per 10 ms | n.A. | n.A. |  |  |
| QRS, per 10 ms | 1.46 (1.13-1.93) | 0.006 | 1.34 (1.02-1.79) | 0.041 |
| Comparison of both ECGs | | | | |
| ΔPR, per 10 ms | n.A. | n.A. |  |  |
| ΔQRS, per 10 ms | 1.11 (0.95-1.32) | 0.196 |  |  |

AF - atrial fibrillation; BMI - body mass index; CAD - coronary artery disease; DPmean - mean transvalvular pressure gradient; LBBB - left bundle branch block; LVEF - left ventricular ejection fraction; NYHA - New York Heart Association.

**Supplement 4** Univariate and Multivariate Predictors for prolonged HV Interval of sinus rhythm patients

|  | Univariate OR (95%CI) | P Value | Multivariate OR (95%CI) | P Value |
| --- | --- | --- | --- | --- |
| Age, years | 1.04 (0.96-1.13) | 0.328 | 1.06 (0.97-1.18) | 0.229 |
| Male sex | 2.89 (1.23-6.99) | 0.016 | 2.17 (0.75-6.47) | 0.156 |
| Body surface area, per 0.1m^2^ | 1.25 (1.05-1.51) | 0.016 | 1.28 (1.01-1.64) | 0.043 |
| Self-expandable | 0.78 (0.33-1.91) | 0.585 |  |  |
| Balloon-expandable | 0.92 (0.20-3.21) | 0.899 | 0.79 (0.13-3.66) | 0.773 |
| Mechanical-expandable | 1.41 (0.52-3.58) | 0.480 | 1.46 (0.47-4.31) | 0.496 |
| ΔPR, per 10 ms | 1.49 (1.19-1.93) | 0.001 | 1.53 (1.20-2.04) | 0.001 |

**Supplement *5:*** Univariate and Multivariate Predictors for prolonged HV Interval of patients in sinus rhythm with additional (to table 3) correction for class III antiarrhythmic medication.

|  | Univariate OR (95%CI) | P Value | Multivariate OR (95%CI) | P Value |
| --- | --- | --- | --- | --- |
| Age, y | 1.04 (0.96-1.13) | 0.328 | 1.08 (0.97-1.20) | 0.164 |
| Male sex | 2.89 (1.23-6.99) | 0.016 | 1.71 (0.54-5.42) | 0.138 |
| Height, cm | 1.04 (0.99-1.10) | 0.128 |  |  |
| Weight, kg | 1.03 (1.00-1.05) | 0.027 |  |  |
| Body surface area, per 0.1m^2^ | 1.25 (1.05-1.51) | 0.016 | 1.32(1.03-1.72) | 0.030 |
| Hypertension | 0.54 (0.22-1.38) | 0.187 |  |  |
| CAD | 1.72 (0.73-4.15) | 0.217 |  |  |
| Dyslipidemia | 1.01 (0.43-2.41) | 0.977 |  |  |
| Diabetes mellitus | 0.64 (0.20-1.77) | 0.420 |  |  |
| Prior myocardial infarction | 0.62 (0.17-1.85) | 0.430 |  |  |
| Prior stroke | 1.50 (0.44-4.52) | 0.484 |  |  |
| Stable angina pectoris | 0.92 (0.37-2.19) | 0.854 |  |  |
| AF | 2.43 (0.97-5.99) | 0.055 |  |  |
| NYHA |  |  |  |  |
| I |  |  |  |  |
| II | 0.56 (0.15-2.41) | 0.409 |  |  |
| III | 0.59 (0.16-2.50) | 0.443 |  |  |
| IV | 1.12 (0.12-8.78) | 0.911 |  |  |
| Previous cardiac surgery | 2.16 (0.42-9.43) | 0.314 |  |  |
| Medication |  |  |  |  |
| Beta-blockers |  |  |  |  |
| …Class Ic |  |  |  |  |
| …Class III |  |  | 17.33(2.93-102.61) | 0.002 |
| Preinterventional echocardiography | | | | |
| DPmean, mmHg | 0.99 (0.97-1.02) | 0.681 |  |  |
| Aortic valve area, per 0.1 mm^2^ | 1.16 (0.92-1.47) | 0.208 |  |  |
| LVEF, % | 1.01 (0.97-1.04) | 0.726 |  |  |
| Baseline ECG | | | | |
| PR, per 10 ms | 1.09 (0.96-1.25) | 0.174 |  |  |
| QRS, per 10 ms | 1.03 (0.87-1.21) | 0.706 |  |  |
| ECG after TAVR | | | | |
| PR, per 10 ms | 1.27 (1.11-1.48) | 0.001 |  |  |
| QRS, per 10 ms | 1.37 (1.03-1.84) | 0.031 |  |  |
| Comparison of ECG before and After TAVR | | | | |
| ΔPR, per 10 ms | 1.49 (1.19-1.93) | 0.001 | 1.51 (1.17-1.94) | 0.001 |
| ΔQRS, per 10 ms | 1.09 (0.91-1.31) | 0.345 |  |  |

**Supplemental 6**: Baseline and procedural data for the three valve types

| Parameter | Valve types |  |  | p-value |
| --- | --- | --- | --- | --- |
|  | *Balloon-expandable*  (n=20)1 | *Self-expandable*  (n=97)2 | *Mechanical-expandable*  (n=34)3 |  |
| Age, years | 82±6 | 82±6 | 82±7 | 0.751 |
| Male sex | 8 (40%) | 37 (38%) | 20 (41%) | 0.949 |
| Height, cm | 167±10 | 166±7 | 165±9 | 0.859 |
| Weight, kg | 73 (64; 92) | 70 (61; 82) | 73 (62; 88) | 0.556 |
| Body surface, m^2^ | 1.94±0.35 | 1.82±0.22 | 1.84±0.25 | 0.612 |
| Hypertension | 17(85%) | 75 (77%) | 22 (65%) | 0.193 |
| CAD | 7 (35%) | 48 (49%) | 18 (53%) | 0.414 |
| Dyslipidemia | 8 (40%) | 55 (57%) | 18 (53%) | 0.393 |
| Diabetes | 8 (40%) | 26 (27%) | 7 (21%) | 0.299 |
| Prior myocardial infarction | 2 (10%) | 18 (19%) | 6 (18%) | 0.651 |
| Prior stroke | 3 (15%) | 15 (15%) | 3 (9%) | 0.622 |
| Angina pectoris | 9 (45%) | 31 (32%) | 12 (35%) | 0.691 |
| Atrial fibrillation | 8 (40%) | 41 (42%) | 10 (29%) | 0.416 |
| NYHA |  |  |  | 0.374 |
| I | 1 (5%) | 9 (9%) | 6 (18%) |  |
| II | 4 (20%) | 42 (43%) | 11 (32%) |  |
| III | 13 (65%) | 41 (42%) | 15 (44%) |  |
| IV | 2 (10%) | 5 (5%) | 2 (6%) |  |
| Previous cardiac surgery | 1 (5%) | 10 (10%) | 1 (3%) | 0.343 |
| Medication |  |  |  |  |
| Beta-blockers | 10 (50%) | 53 (54%) | 14 (41%) | 0.400 |
| …Class III | 1 (5%) | 10 (10%) | 1 (3%) | 0.343 |
| **Pre-procedural echocardiography** |  |  |  |  |
| DPmean, mmHg | 52 (38; 67) | 45 (37; 55) | 51 (44; 61) | 0.031 |
| Aortic valve area, cm^2^ | 0.8±0.3 | 0.7±0.2 | 0.7±0.2 | 0.842 |
| LVEF, % | 59 (45; 60) | 55 (45; 60) | 60 (45; 61) | 0.905 |
| **Baseline ECG** |  |  |  |  |
| PR interval, ms | 199 (171; 223) | 178 (157; 200) | 171 (151; 202) | 0.145 |
| QRS duration, ms | 105 (94; 116) | 95 (87; 112) | 97 (90; 107) | 0.309 |
| **Post-TAVR ECG** |  |  |  |  |
| HV interval, ms | 54±11 | 52±12 | 53±11 | 0.576 |
| PR interval, ms | 215 (178; 238) | 196 (169; 213) | 190 (160; 224) | 0.197 |
| QRS duration, ms | 142 (135; 154) | 146 (140; 157) | 146 (133; 158) | 0.610 |
| ΔPR, ms | 6 (2.8; 16) | 9 (0; 18) | 12 (3;20) | 0.620 |
| ΔQRS, ms | 42 (27; 52) | 51 (34; 60) | 48 (29;63) | 0.287 |
|  |  |  |  |  |

Data are presented as mean±SD or median (interquartile range) for continuous variables and as n (%) for categorical variables.

BMI - body mass index; CAD - coronary artery disease; DPmean - mean transvalvular pressure gradient; LBBB - left bundle branch block; LVEF - left ventricular ejection fraction; NYHA - New York Heart Association.
